# SIMR foci are found in the progenitor germ cells of *C. elegans* embryos

**DOI:** 10.17912/micropub.biology.000374

**Published:** 2021-02-22

**Authors:** Celja J. Uebel, Kevin I. Manage, Carolyn M. Phillips

**Affiliations:** 1 Department of Biological Sciences, University of Southern California, Los Angeles, California, USA

## Abstract

RNA interference is a widely conserved mechanism of gene regulation and silencing across eukaryotes. In *C. elegans*, RNA silencing is coordinated through perinuclear nuage containing at least four granules: P granules, Z granules, *Mutator* foci, and SIMR foci. Embryonic localization of these granules is known for all except SIMR foci. Here we establish that SIMR foci first appear at the nuclear periphery in the P_4_ germline blastomere and become numerous and bright in the Z2 and Z3 progenitor germ cells. This timing coincides with the appearance or de-mixing of other germline granules, providing further evidence for coordinated germ granule reorganization.

**Figure 1. SIMR foci are numerous and bright in the Z2/Z3 progenitor germ cells:**
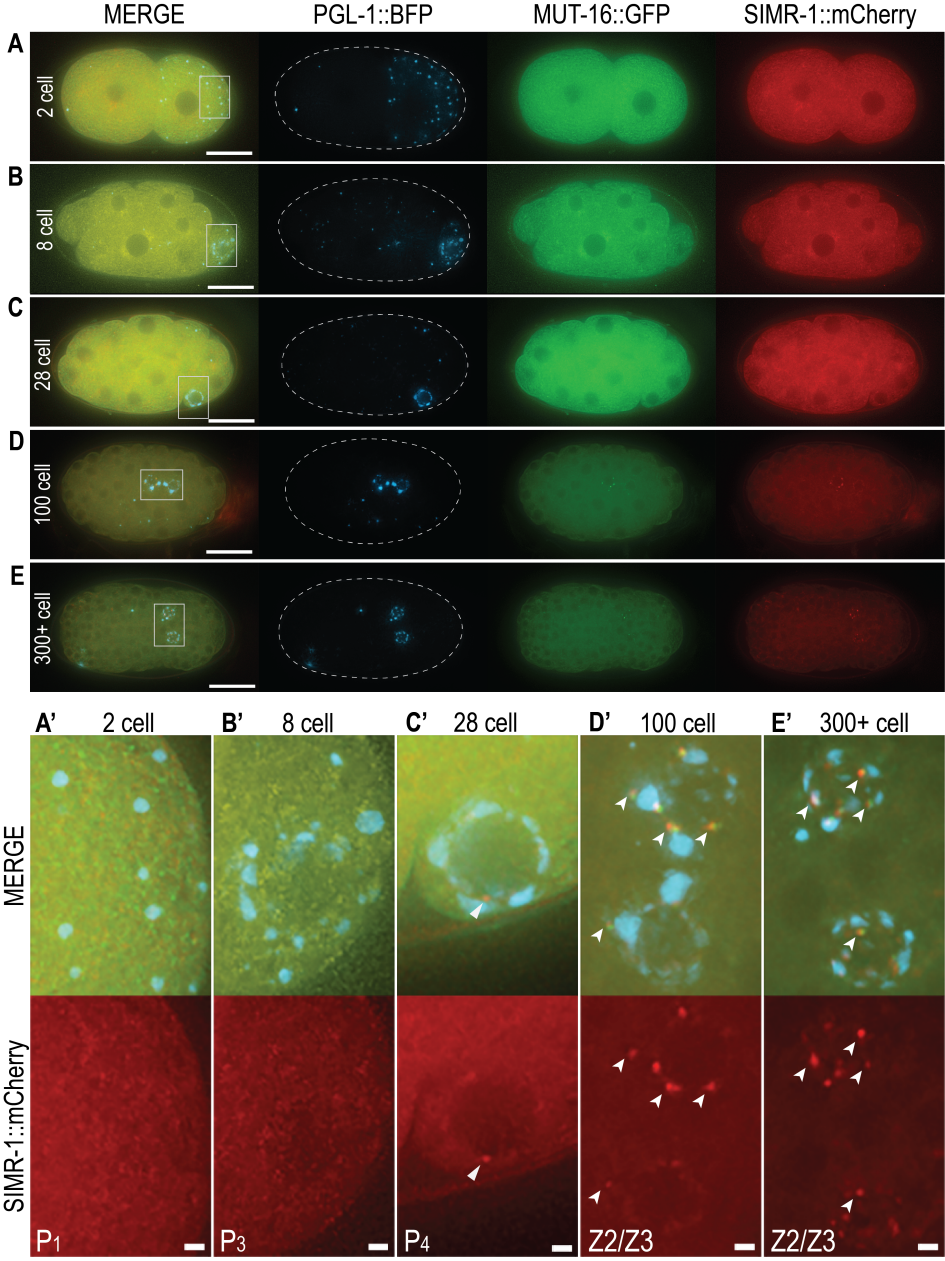
**A-E**: Representative live images of embryos expressing PGL-1::BFP (blue), MUT-16::GFP (green) and SIMR-1::mCherry (red) to visualize P granules, *Mutator* foci, and SIMR foci, respectively. Scale bars, 15 µm. For each stage at least 3 embryos were observed. **A’-E’**: Inset from boxed outline in A-E merge, highlighting the P lineage and progenitor germ cells. Scale bars, 1 µm. **C’**: Triangle arrowhead indicates early SIMR focus. **D’-E’**: Notched arrowheads indicate bright, numerous SIMR foci (red) interacting with *Mutator* foci (green) and P granules (blue).

## Description

Multiple condensates occupy the perinuclear space of *C. elegans* germ cells, where they coordinate RNA surveillance to ensure proper gene expression (Lev and Rechavi 2020; Sundby *et al.* 2021). The most well-studied of these condensates are P granules, phase-separated germ granules required for maintenance of germ cell identity and fertility (Kawasaki *et al.* 1998; Updike *et al.* 2014). P granule morphology and localization is well documented in *C. elegans* development (Strome *et al.* 1982). Adjacent to P granules are *Mutator* foci, which are nucleated by MUT-16 and required for the amplification of small interfering RNAs (siRNAs) to create a robust and heritable silencing signal (Phillips *et al.* 2012). During development, faint *Mutator* foci are occasionally seen in the P_4_ germline blastomere of 30-cell embryos, but are most robust and numerous in the Z2 and Z3 progenitor germ cells (PGCs) of 100-cell embryos (Uebel *et al.* 2020). A third germline condensate, Z granules, are situated between P granules and *Mutator* foci and facilitate transgenerational epigenetic inheritance of silencing signals. Z granule components ZNFX-1 and WAGO-4 colocalize with P granules in early embryos, but begin to de-mix from P granules in the Z2/Z3 PGCs to form separate Z granule condensates (Wan *et al.* 2018). Lastly, recently discovered SIMR foci also intimately localize within this cluster of germline granules. SIMR-1, a key component of SIMR foci, is a Tudor domain protein that mediates production of secondary siRNAs for piwi-interacting RNA (piRNA)-targeted mRNAs (Manage *et al.* 2020). While P granule, Z granule, and *Mutator* foci localization through embryonic development has been previously described, the embryonic appearance of SIMR foci is not known.

Here we use endogenously tagged SIMR-1::mCherry to investigate the embryonic onset of SIMR foci. We further visualize embryonic P granules with PGL-1::BFP and *Mutator* foci with MUT-16::GFP to compare fluorescence and interaction with SIMR foci. Live imaging of embryos reveals diffuse cytoplasmic expression of SIMR-1 in all stages (Figure 1A-E), similar to previous observations of MUT-16 expression (Uebel *et al.*. 2020). Because SIMR foci are present in the germlines of adult hermaphrodites and localize adjacent to P granules, we focused our analysis on the germline blastomeres and progenitor germ cells of embryos (Figure 1A’-E’). In the 2-cell embryo, P granules segregate to the posterior P_1_ germline blastomere, yet no punctate SIMR foci are present (Figure 1A, A’). Similarly, no SIMR foci are found in 8-cell embryos as P granules begin associating with nuclear pores in the P_3_ germline blastomere (Figure 1B, B’). The 28-cell embryo yields the first observable SIMR focus adjacent to perinuclear P granules in the P_4_ germline blastomere (Figure 1C, C’). While we consistently observe SIMR foci in 28- to 50-cell embryos (n = 3), these foci are few and faint. Around the 100-cell stage, the P_4_ cell gives rise to the Z2 and Z3 PGCs, and it is here that we reliably observe bright and numerous SIMR foci (Figure1D, D’). These bright foci also persist in the Z2/Z3 of late-stage embryos of 300 or more cells (Figure1E, E’). Our data reveals the previously unknown embryonic appearance of SIMR foci.

Consistent with their localization in adult germ cells, embryonic SIMR foci appear adjacent to both P granules and *Mutator* foci. Interestingly, the appearance of fewer, faint SIMR foci in the P_4_ cell and more numerous, bright SIMR foci in the Z2/Z3 PGCs is similar to the timing of *Mutator* foci formation in embryos (Uebel *et al.*. 2020). Both the de-mixing of Z granules and the appearance of robust *Mutator* foci and SIMR foci in the PGCs correlates with the onset of embryonic germline transcription (Seydoux and Dunn 1997, Wan *et al.* 2018, Uebel *et al.* 2020). Taken together, this observation suggests that the arrival of newly produced mRNAs in the Z2/Z3 PCGs may necessitate or facilitate the coordinated reorganization of germ granule components for efficient RNA surveillance.

## Methods

**Microscopy**

Worms were grown at 20°C according to standard conditions (Brenner 1974). Gravid adult *C. elegans* were dissected in 10 µL M9 to expose embryos and mounted on a fresh 2% agarose pad for live imaging. At least 3 embryos were observed for each stage. All images were acquired with a DeltaVision Elite (GE Healthcare) microscope using a 60x N.A. 1.42 oil-immersion objective. Ten 0.2-micron Z stacks were compiled as maximum intensity projections and pseudo-colored using Adobe Photoshop to create each image. The same exposure, acquisition, and pseudo-coloring settings were used for each image.

## Reagents

**USC1401** *simr-1(cmp15[simr-1::mCherry::2xHA]) mut-16(cmp3[mut-16::gfp::3xFLAG]) I; pgl-1(cmp226[pgl-1::bfp::3xFLAG]) IV.*

**Strain Construction**

USC1401 was created by crossing USC1269 (*pgl-1(cmp226[pgl-1::bfp::3xFLAG])*) (Uebel and Phillips 2019) and USC774 (*simr-1*(*cmp15[simr-1::mCherry::2xHA]) mut-16(cmp3[mut-16::gfp::3xFLAG]) I; unc-119(ed3) III*) (outcrossed) (Manage *et al.* 2020). All strains are available upon request.
